# The Twenty-fifth Volume

**Published:** 1868-01-01

**Authors:** 


### *EDITORIAL.*

---

WITH this number commences the Twenty-Fifth Annual Volume of this Journal, and it is with unmingled pleasure we undertake both its enlargement and more frequent issue. In these times the monthly bears about the same relation to the real wants of the profession that the semi-annual did a few years since. When the means of communication between the different parts of the country and the world were more limited, the thoughts of men were slowly concentrated, and the Medical Journal was obliged to depend on the professional contributions of those within its own limited sphere. Now the learned, observant and experienced, every where about the globe, can speedily bring together whatever of the new, and possibly true, they have gathered up, and cast them into the crucible of universal analysis. The Present becomes its own sharply interrogating and impartially judging Posterity.

The Profession, having thrown off the trammels of old-time dogmatism, can not wait the slow processes of antiquity. It must move in the van of the grand march of Progress which distinguishes the age. It is needless to say that, for this purpose, the means of communication between its members should be facilitated to the greatest possible extent.

For this, among other reasons, the JOURNAL advances from the monthly to the semi-monthly—anticipating the not distant future when this will be replaced by the weekly, and within the next quarter centennial period by the daily.

If it were not for the editorial horror of elongated articles, in this age of telegraphic conciseness, there would be more written here on this point, but why "carry coals to Newcastle?" This semi-monthly is already an established fact, and its success as such placed beyond contingency.

The Editor congratulates his subscribers, both old and new, on the advanced position, and extends to them the salutations of the New Year.

